# Automated Optimization of a Multistep, Multiphase Continuous Flow Process for Pharmaceutical Synthesis

**DOI:** 10.1021/acssuschemeng.4c05015

**Published:** 2024-10-03

**Authors:** Sarah L. Boyall, Holly Clarke, Thomas Dixon, Robert W. M. Davidson, Kevin Leslie, Graeme Clemens, Frans L. Muller, Adam D. Clayton, Richard A. Bourne, Thomas W. Chamberlain

**Affiliations:** † Institute of Process Research and Development, School of Chemistry & School of Chemical and Process Engineering, University of Leeds, Leeds LS2 9JT, England; ‡ Dr. Reddy’s Laboratories (EU), 410 Science Park, Milton Road, Cambridge CB4 0PE, U.K.; § Chemical Development, Pharmaceutical Technology & Development, Operations, AstraZeneca, Macclesfield SK10 2NA, U.K.

**Keywords:** flow chemistry, telescoping, multistep, heterogeneous catalysis, three-phase, multiphase, self-optimizing algorithms

## Abstract

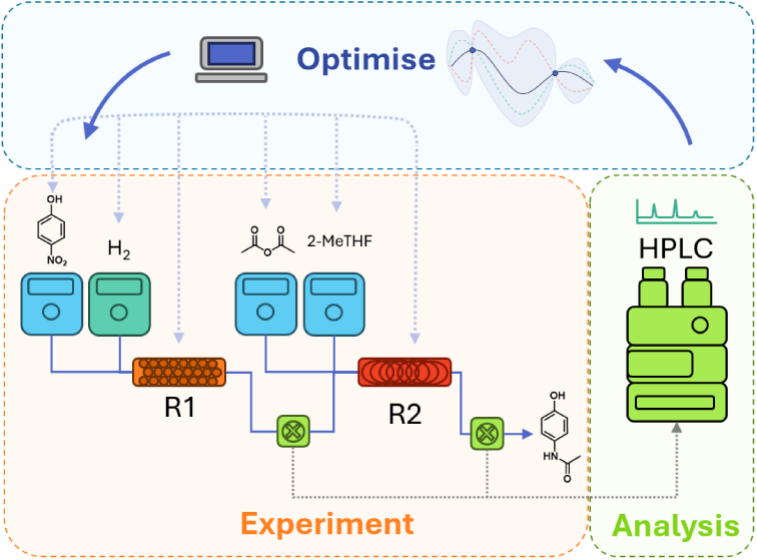

Flow synthesis is becoming increasingly relevant as a sustainable and safe alternative to traditional batch processes, as reaction conditions that are not usually achievable in batch chemistry can be exploited (for example, higher temperatures and pressures). Telescoped continuous reactions have the potential to reduce waste by decreasing the number of separate unit operations (e.g., crystallization, filtration, washing, and drying), increase safety due to limiting operator interaction with potentially harmful materials that can be reacted in subsequent steps, minimize supply chain disruption, and reduce the need to store large inventories of intermediates as they can be synthesized on demand. Optimization of these flow processes leads to further efficiency when exploring new reactions, as with a higher yield comes higher purity, reduced waste, and a greener synthesis. This project explored a two-step process consisting of a three-phase heterogeneously catalyzed hydrogenation followed by a homogeneous amidation reaction. The steps were optimized individually and as a multistep telescoped process for yield using remote automated control via a Bayesian optimization algorithm and HPLC analysis to assess the performance of a reaction for a given set of experimental conditions. 2-MeTHF was selected as a green solvent throughout the process, and the heterogeneous step provided good atom economy due to the use of pure hydrogen gas as a reagent. This research highlights the benefits of using multistage automated optimization in the development of pharmaceutical syntheses. The combination of telescoping and optimization with automation allows for swift investigation of synthetic processes in a minimum number of experiments, leading to a reduction in the number of experiments performed and a large reduction in process mass intensity values.

## Abstract 2

Telescoping self-optimizing continuous flow chemistry efficiently found optimum reaction conditions and reduced PMI values by half for a multiphasic process.

## Introduction

Traditionally, the synthesis of active pharmaceutical ingredients (APIs) has been carried out in multiple, single-step batch reactions, where one compound is synthesized and subsequently purified and isolated before being used in the next step of the process. These steps can be located at different manufacturing sites in different countries, resulting in increased shipping and transportation costs.^1−3^ This can be costly, time-consuming, and potentially dangerous when reaction intermediates are particularly hazardous and need to be transported between facilities. An alternative approach would be to employ telescoped continuous flow chemistry, where each step is directly flowed into the next step in a single environment. Flow chemistry offers significant benefits over traditional batch chemistry, with better process control, improved heat and mass transfer, and often smaller plant footprints, allowing multiple steps to be carried out in smaller facilities. Subsequent telescoping of reaction steps leads to a great reduction in the unit operations required in the overall synthesis, as a single purification can often be performed at the end of multiple steps instead of after each individual reaction.^3−7^ Reducing the number of reaction steps can lead to a reduction in operating costs, making this method for synthesis more viable for underprivileged regions globally and satisfies a number of the 12 principles of green chemistry.^8−10^ Telescoping flow systems have the potential to result in fast, on demand drug synthesis. With engineering advancements, this could reduce plant size while continuing to make APIs on a kg day^–1^ scale. If achieved, this would improve drug accessibility and affordability worldwide.

Self-optimizing algorithms have been used in conjunction with automated closed-loop computer-controlled systems and process analytical technology (PAT), to explore a predefined experimental design space, and optimize a chemical process for a desired objective (for example, yield or reaction mass efficiency).^11−13^ Based on previous experiments and objective values generated from PAT, the algorithm will suggest a new set of experimental conditions to maximize or minimize the selected objective. When coupling telescoped continuous flow processes with automation and self-optimizing algorithms, human error and interaction time can be minimized, and self-optimization often leads to fewer experiments needing to be performed, reducing solvent use and operational costs.^14^ Single step, single- and multiobjective optimizations have seen increasing attention in the past few decades and have been widely reported in the literature.^15−21^ However, the application of self-optimizing algorithms to multiple steps simultaneously is less common, with many multistep examples being optimized in the more traditional one factor at a time approach.^22−26^ Making use of multiple PAT technologies including flow NMR and FTIR, Sagmeister et al. optimized a complex two-step, seven variable system in 85 experiments. They developed a modular platform that harnessed the power of rapid spectroscopic measurements processed by chemometric models to fully explore the complicated design space, achieving a high space-time yield (STY).^27^ By optimizing to minimize equivalents of reagents while maximizing yield, they found it was possible to use substoichiometric amounts of reagents to give a greener process while maintaining high yields and STY. Clayton et al. reported the use of Bayesian optimization algorithms for a telescoped process, where a single high-performance liquid chromatography (HPLC) was used for accurate quantification of reaction components and optimization of two reaction steps simultaneously by multipoint sampling.^28^ This method made multistep analysis and optimization more accessible for a wider number of research groups by limiting the number of expensive PAT needed to gain a process understanding of the entire system.

Integrating multiphase chemistry into continuous flow can be challenging but is extremely advantageous, as solid catalysts can be introduced simply using a packed bed or coated reactors. This benefits from the ease of catalyst separation, leading to a reduction in the loss of precious metals and a decrease in the processing time used to filter off spent catalyst. Therefore, packed bed reactors are an excellent way to integrate heterogeneous catalysis into flow chemistry. Due to the improved mass transfer of flow, using gaseous reactants such as hydrogen can be highly beneficial, is less problematic than in batch, and has additional safety benefits of working with comparatively smaller quantities of gases at any one time, avoiding large headspaces of hydrogen.^29,30^ However, challenges in telescoping such reactions arise with the separation of multiple phases for accurate sampling and telescoping at pressure, and catalyst deactivation of solid catalyst particles is a major concern for automated optimizations.^31^ Kappe et al. reported an investigation into a single step heterogeneous hydrogenation reaction, evaluating the tolerance and stability of the catalyst to a wide range of functional groups in a systematic screening platform. This paper highlights the need to understand catalyst deactivation (reversible and irreversible) as well as monitoring it for accurate optimization of reaction conditions.^32^ Nambiar et al. showed the use of multiobjective optimization algorithms to optimize continuous and discrete variables for a three-step telescoped process including a S_N_Ar, heterogeneous nitro reduction, and amide coupling reaction.^31^ However, due to issues with catalyst deactivation when telescoping to the second step (nitro reduction), the S_N_Ar reaction was optimized in a separate campaign. To maintain catalyst stability during the subsequent telescoped reaction, the temperature of the catalyst bed was set to 125 °C, and only the equivalence of the nitro acid starting materials was optimized for the nitro reduction.

Herein, we describe a comparison of the self-optimization of a single vs multistep process, which includes a multiphasic heterogeneously catalyzed hydrogenation, to highlight the benefits of optimizing both reaction steps simultaneously. To compare the different modes for optimization, a two-step process to make paracetamol, a nonsteroidal anti-inflammatory painkiller found on the World Health Organization’s (WHO) list of essential medicines, was selected.^33^ Due to its important nature, on demand manufacture of this medicine is globally critical, and multistep telescoped continuous flow processes can provide a low-cost small footprint manufacturing method.

## Results and Discussion

**Scheme 1:**
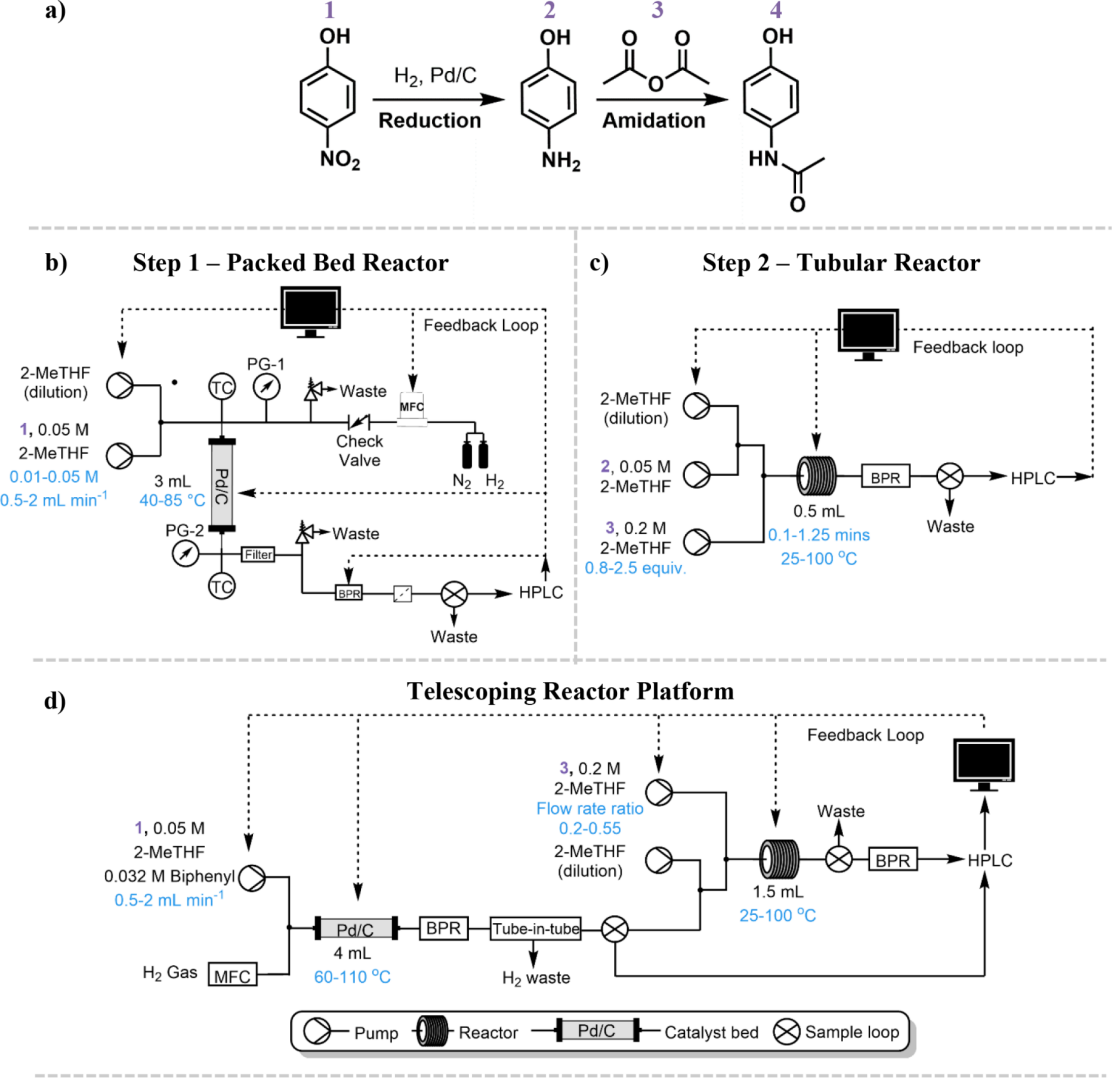
Reactor Setups for Each Reaction Step (a) Scheme for two-step process to make paracetamol where 1—4-nitrophenol, 2—4-aminophenol, 3—acetic anhydride, and 4—acetaminophen. (b) Packed bed reactor setup for step one—heterogenous hydrogenation. (c) Heated coiled tubular reactor setup for step two—amidation. (d) Combined telescoping reactor for two-step production of paracetamol. The variables and bounds are highlighted in each reactor setup in blue text. More information on systems can be found in Section S1.

A comparison of the two methods of optimization for the multistep synthesis of paracetamol is outlined here. The two reaction steps of interest (highlighted in Scheme 1a) were an initial three-phase heterogeneous hydrogenation of 4-nitrophenol **1** (involving a solid catalyst, gaseous hydrogen, and liquid reactants) followed by an amidation of the product of step one, 4-aminophenol **2** to make the final product acetaminophen **4**. The heterogeneous hydrogenation was conducted in a packed bed reactor previously reported for catalyst scale-up performance testing by Boyall et al.^34^ The amidation was performed in a plug flow perfluoroalkoxy alkane (PFA) tubular reactor. Both reactor setups are shown in Scheme 1b,c and are outlined further in Section S1. Typically, solvents such as alcohols are chosen for heterogeneous hydrogenations of nitro compounds,^35^ but when combining these steps, any alcohols would react with the acetic anhydride in step two, forming undesired side products and deactivating the reagent. Derived from biomass, 2-methyltetrahydrofuran (2-MeTHF) has been successfully used as a solvent in biological and lab scale applications and is frequently used as a greener alternative to dichloromethane and tetrahydrofuran.^36^ Therefore, using the solvent selection guide in an aim to make the process as green and sustainable as possible, 2-MeTHF was chosen as an appropriate solvent for both reaction steps.^37^ It is beneficial to maintain the same solvent throughout a multistep process as switching solvents is energy and waste intensive. Catalyst deactivation was monitored throughout the optimization by running a standard set of reaction conditions, repeated every fourth experiment of the campaign sampled offline, and does not count toward the overall optimization. This was to ensure that any change in the yield of the reaction was due to the conditions applied to the system and not because of a change in catalyst activity.

Both individual reaction steps were first optimized separately in a one-step at a time (OSAT) approach, using a Bayesian optimization algorithm with an adaptive expected improvement acquisition function (BOAEI) previously reported by Clayton et al.^28,38^ The algorithm dynamically changes/controls the ratio between exploration of the design space and exploitation of the optimum conditions, aiming to minimize the number of experiments required, while sufficiently exploring the design space to find the global optimum. This negates the need to predefine the trade-off between exploration and exploitation for the algorithm, removing bias from the optimization. The termination of each optimization was decided when no further improvement in the yield was seen after at least five experiments, corresponding to a plateau in the objective function (see Figure S6 in Section S2). While in this work the optimizations were manually terminated, it could be possible to integrate the autonomous termination of the algorithm. One possible termination criterion could be when there has been no substantial improvement in the objective over a specified number of iterations. Alternatively, the confidence bounds of the surrogate model could be monitored, and once the uncertainty in the model predictions is sufficiently low (a predefined value), the algorithm would terminate. These approaches could be combined to provide more robust termination criteria. However, due to the complexity of this system, having a human operator present enabled rapid identification of any unexpected behavior or anomalies that could potentially misguide the algorithm. An example of such occurrence may be in the missampling of a reaction step due to a failure in the separation of the gas and liquid phases. This would lead to a blank or low concentration result from the HPLC chromatogram that may mislead the algorithm and cause it to suggest incorrect future experiments, possibly leading to the optimum conditions being missed. In this instance, the optimization would be stopped, the previous experiment would be discounted, and the reaction would be restarted again from the last correctly performed optimization result.

**Figure 1:**
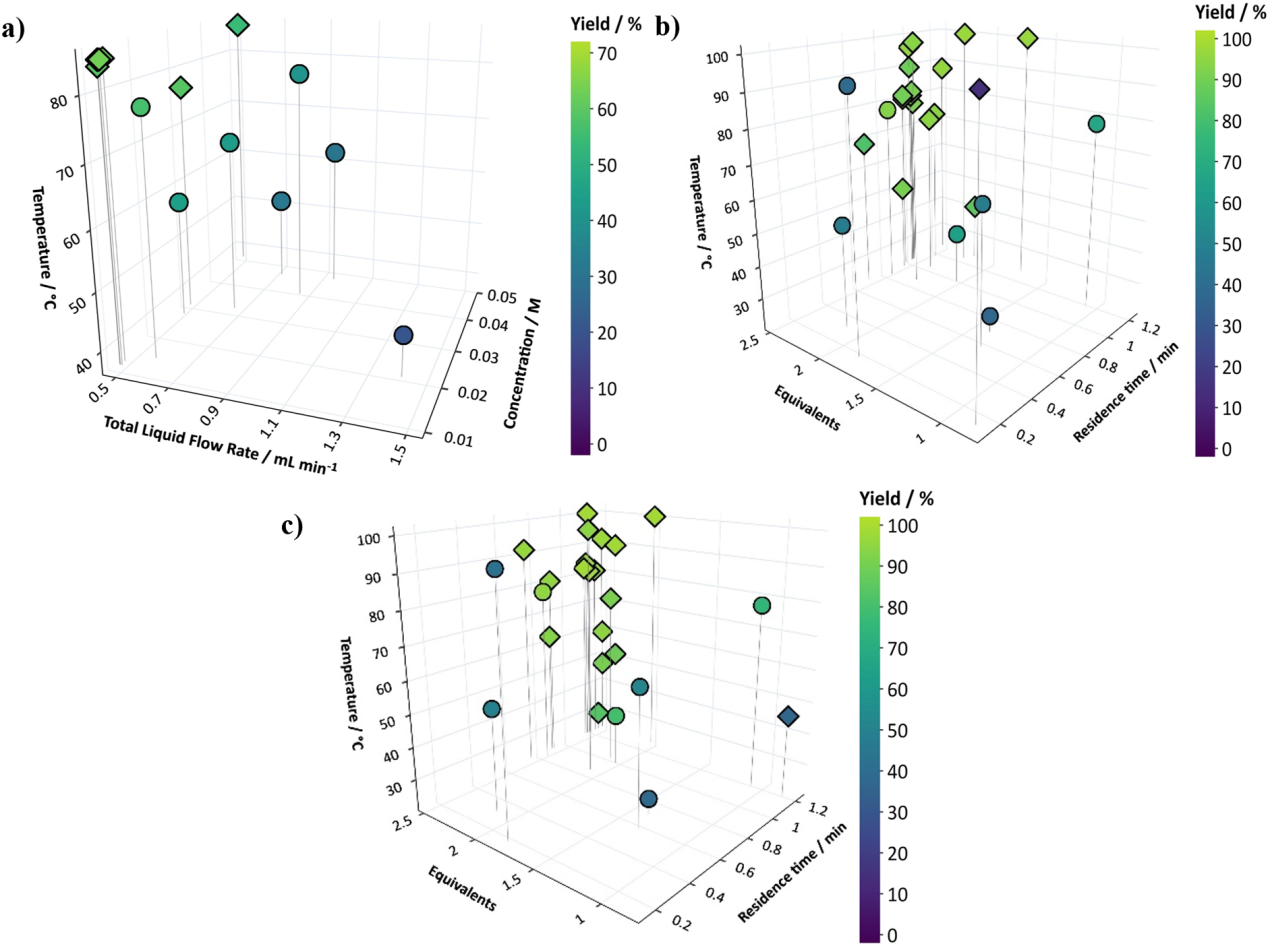
Self-optimization results for single step reactions where ○—LHS experiments and ◊—refinement experiments. (a) Optimization for step one—heterogeneous hydrogenation. (b) Optimization for step two—amidation. (c) Optimization of step two—amidation—with a reservoir created from the outlet of step one at the optimum conditions, where the yield refers to the consumption of aniline in the reservoir (65%), and not the overall yield of the two-step process.

A single objective optimization for yield of the heterogeneous hydrogenation was performed using the BOAEI algorithm, the results are shown in Figure 1a, and all optimization data are summarized in Section S3 (all quoted yields were based on HPLC data). Optimization graphs were plotted using Plotly, and more information can be found in Section S4. This found optimal conditions for step one, yielding 65% of 4-aminophenol product (with the remaining 35% left as unreacted starting material) at low liquid flow rates equating to long residence times, high temperatures, and low starting material concentrations, after only nine experiments, which includes seven initial conditions from the Latin hypercube sampling (LHS). LHS was used as the space filling design to initialize the algorithm, as it generates a near random sample of parameter values that ensures a good spread of initial conditions are explored across the entire design space.^39^ Requiring only two algorithm conditions to maximize the overall yield, this shows the efficiency of BOAEI in finding optimal reaction conditions. Long residence times and high temperatures could be used to achieve high yields, in this case, owing to no competing side reactions or catalyst deactivation observed for the 14-experiment optimization, with similar trends reported by Bukhtiyarova et al.^40^ Observation of molecule **2** (step one product) over time showed a color change from colorless to dark brown solution over a period of 3 h, due to oxidation of the product, further highlighting the benefits of performing paracetamol synthesis in a telescoped process, as the stability of molecule **2** would be less of a concern.

A single objective optimization for step two was also performed, optimizing for yield while varying temperature, equivalents of **3** and the residence time of the reaction. High yields of >85% were achieved in 10 out of the 25 experiments performed and an optimum yield of 97% at high equivalents, high temperatures, and a range of residence times was found after 12 experiments. The main variable that affected yields in this reaction was the equivalents of **3**, with most of the optimum points requiring an acetic anhydride equivalents of >2 (Figure 1b), which aligns with the literature as often neat acetic anhydride is used for the synthesis of **4**.^41^ This could be due to some acetic anhydride being lost in a reaction to water present in the reaction solution (as the reaction is not under anhydrous conditions) to form acetic acid during the acylation; therefore, an excess is required to achieve full conversion to the acetaminophen product. Too much acetic anhydride can lead to the formation of the byproduct 4′-acetoxyacetanilide, where the molecule is acylated at both the OH and NH_2_ functional groups.^42,43^ However, this was not seen within the variable bounds set for this optimization. In Section S5, the acetic anhydride equivalents were shown to be the most strongly correlated with the yield of reaction according to the Matern 5/2 kernel length scale—N.B. The lower the kernel length scale value, the more significant the variable is to the optimization. To improve process metrics, a shorter residence time would be preferred, to increase the throughput of the reaction while maintaining high yields. This can be found at residence times of 0.95 min.

A second optimization protocol for step two was performed, which aimed to determine whether having unreacted **1** starting material, minor impurities, and/or side products from an incomplete reaction of step one had any impact on the optimization of the second reaction step. Control experiments were initially performed to assess the effect of unreacted **1** at the optimal conditions found for step two with a reservoir containing commercially bought starting materials (with a 50:50 ratio of **1**:**2**). At the optimal reaction conditions, it was found that there was no effect of unreacted **1** on the yield of the reaction, with the optimum remaining at 98% yield of **4**, with no other impurities formed. The optimal conditions, found in the optimization of step one, were used to create a reservoir of **2** (yield of 65%) and **1** (35%). This was then used as the initial starting solution, i.e., a model, intermediate mixture (IM), for the optimization of step two to investigate the effects of other conditions within the design space on the yield and side product formation of the reaction. No other side products were found due to unreacted starting material **1** from step one during this 25-experiment optimization and the optimum conditions found (see Figure 1c) were the same for both individual optimizations for this step and are summarized in Section S6.

The final challenge was to combine both reactor platforms to perform a fully telescoped two-step single objective optimization. For this to be fully autonomous, a gas–liquid separator was required to remove the gas phase between the two reactors. A tube-in-tube separator previously reported by Harding et al.^44^ was used to separate the gas and liquid phases at pressure (7 bar). The separator is made of a porous ePTFE tube encased in a stainless-steel tube, which allowed only the liquid phase to permeate through the tubing and be separated from the gas phase (more information is detailed in Section S7). This was important, as it would ensure that only the liquid phase was sampled into the HPLC and the residence times in the second reactor remained unaffected by the gas flow. A multipoint sampling system was employed to sample from both reactors into a single HPLC.^28^ This allowed the relationship between all four variables and the yields and selectivity of both reactions to be understood fully, while minimizing the amount of inline analytical equipment needed. A sampling valve was connected to the outlet of each reactor and daisy-chained in loop with the HPLC. Each valve was set to sample as its corresponding reaction step reached steady state, providing sequential analysis of both steps on a single chromatogram, which simplified the autonomous peak interpretation (see Section S8 for example chromatograms). The original six min OSAT HPLC method was doubled to create a 12 min method time, where R1 was sampled at time zero minutes, and R2 was sampled six min into the method. More details on the multipoint sampling system can be found in Section S1.

**Figure 2:**
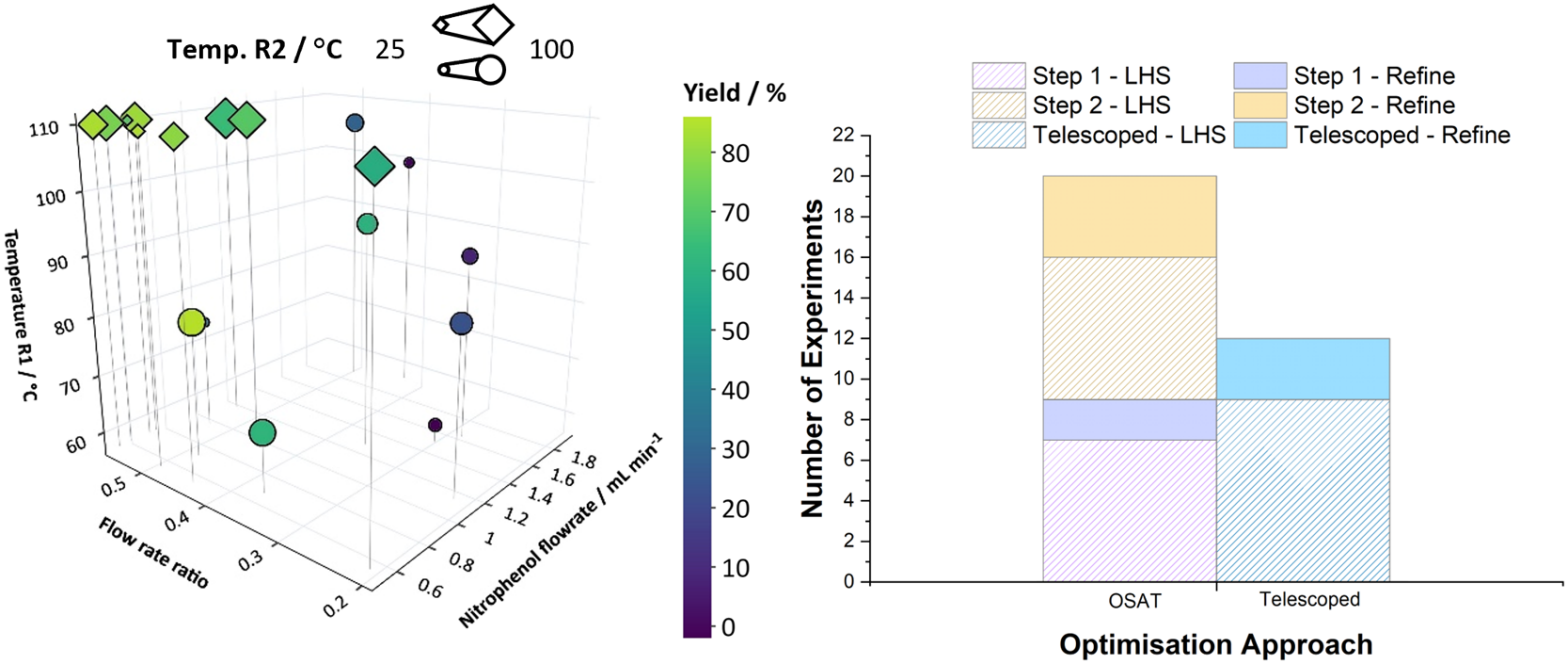
Self-optimization data for the telescoped, two-step system where ○—LHS experiments and ◊—refinement experiments. (left) Comparison of experiment number and maximum yield achieved chronologically in each optimization (right).

To increase the yield of the first step, a greater catalyst amount was used (0.9 g previously used, increased to 1.5 g), and the four variables chosen to be optimized were: (i) temperature of reactor one (R1), (ii) temperature of reactor two (R2), (iii) liquid flow rate of **1** pump, and (iv) flow rate ratio of the **1** pump to the **3** pump (which changed the equivalence of **1** with respect to the product **2**). To achieve the equivalence bounds of **3** in R2 and maintain the same flow rate ranges of **1** in R1 as investigated in the OSAT optimizations, the reactor volume of R2 was increased from 0.5 to 1.5 mL. This was essential to achieve accurate flow rates that were within the operational parameters of the pumps. Concentration was not included in the final two-step telescoped optimization, as it was found to have the least impact on the OSAT optimization of step one (largest kernel length scale—see Section S5). In other telescoped systems, where concentration significantly impacts the optimization objective, concentration could be varied using dilution pumps; however, this would increase the complexity of the system, affect the residence time of the reactor, and dilute any subsequent reactions downstream. In the case of telescoped systems, therefore, it would require careful consideration. Due to the difficulty of decoupling the residence time of R2 in the multistep telescoped system, it was also not investigated in the telescoped optimization. With both systems combined into one reactor setup (Scheme 1d), a four variable, two-step, multiphase optimization was performed, and the results are shown in Figure 2.

Figure 2 shows an optimum yield of 85% was achieved for both steps combined after only 12 experiments (nine initial conditions from LHS, three refinement) with the BOAEI algorithm efficiently exploring the design space and focusing on exploitation after the LHS to find the optimum conditions on shorter time scales. Increasing the number of variables being optimized increases the number of initial conditions required from seven to nine during the LHS (the number of initial conditions is equal to 2*n* + 1, where *n* is the number of variables). The optimum conditions were found at high temperatures of R1, high flow rate ratio (high equivalence of **3**), and low liquid flow rate of the **1** pump relating to a long residence time. After experiment nine, the temperature of R1, flow rate of the **1** pump, and the flow rate ratio were kept constant by the algorithm at the optimal conditions for subsequent reactions, while only changing the temperature of R2. In both single step optimizations for step two, 100 °C was found to be the optimal temperature, which is higher than was found during the telescoped optimization. However, the algorithm explored this variable extensively during all optimizations and many high yielding points were found at a range of temperatures. The calculated kernel length scale values (Section S5) for the telescoped optimization indicated that the R2 temperature had the least impact on the overall yield of the combined two-step process (high kernel length scale value) which coincides with the same finding when optimizing step two, where it was also the least important factor in both the OSAT and IM optimizations. The graph in Figure 2 shows the number of experiments each optimization protocol required to find the optimum conditions of the multistep process, with a combined number of 20 experiments needed to optimize via the OSAT approach, reduced to 12 in the telescoped approach (also shown in Table 1). Only three experiments were required after the LHS for the telescoped process to optimize, reducing the time and resources required to optimize this two-step reaction. Deactivation of the heterogeneous catalyst was also monitored with repeat reactions taken every fourth experiment, with minimal loss in activity observed over the course of the optimization (<5%). The optimization was stopped after 18 experiments, when significant deactivation of the catalyst was suddenly observed (see Section S9 for deactivation data). The optimization was performed over 40 h, with a shutdown procedure implemented in between the consecutive days, which led to a small increase in activity each time (5%). It is likely that the operational lifetime of the catalyst could be extended if periodic reactivation at high temperatures with solvent was performed. At the optimum conditions found in the multistep optimization, over a 40 h operating period, a total of 7.6 g of product could be produced using this relatively small scale setup. This throughput could be increased through, scale-up, scale out, or numbering up of the reactors.

**Table 1:** Comparison of PMI Values and Number of Experiments Needed to Reach the Optimum Reaction Conditions for the Single Step and Telescoped Optimizations

reaction step	total mass/g h^–1^	mass of product/g h^–1^	PMI with 20% solvent mass	% of total mass from solvent	no. of expt. to reach optimum
OSAT—step 1	26.9	0.021	299	96	9
OSAT—step 2	21.6	0.072	61	99	11
OSAT—combined	39.9	0.044	205	97	20
telescoped	44.8	0.190	56	95	12

In order to objectively evaluate the optima determined by both OSAT and telescoping approaches for the optimization of multistep processes, process mass intensity (PMI) values were calculated at the optimum conditions of each optimization step.^45^ This metric is used to evaluate the greenness of the process by taking into account the total amount of material used to produce a mass of product and was chosen to account for the methodology changes. The yield could not be directly compared because the catalyst loading and reactor volume changed during the adaptation of the systems to a fully telescoped process, so PMI was used to account for the extra mass and volume and is calculated using eq 1.
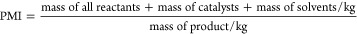
1

A PMI value of one is optimal as it means that everything used in the process is incorporated into the product, and this metric was selected by the Green Chemistry Institute Pharmaceutical Roundtable as their preferred mass-based green metric.^14^ The pharmaceutical industry is taking an active role in reducing PMI in API synethsis, which was exemplified by the report of a 23% reduction in PMI for AstraZeneca’s late stage project portfolio in 2022.^46^ In this work, the PMI values are much higher than many examples in the literature due to the reagents being at a low concentration, to ensure good solubility of the reactants in 2-MeTHF. Therefore, PMI values for 20% mass of 2-MeTHF were calculated as some solvent would likely be able to be recycled from process to process in a “best case” scenario, shown in Table 1.^47^ When comparing these values to solution based literature examples of a two-step paracetamol synthesis, Geib et al. reported a PMI value of 43, which is comparable to the proposed telescoped process.^48^ However, their value does not account for the separation and work up steps associated with multistep processes, which would likely lead to a much higher PMI value. A PMI value was also calculated for the OSAT step one and step two processes if they were combined into a telescoped process under their respective optimal conditions. Here we show that the PMI values for the single step OSAT reactions could be significantly reduced when performing a telescoped process, largely due to a reduction in solvent use in the telescoped system. In typical single step reactions, the products would likely need to be purified, isolated, and crystallized at the end of every step to be used in subsequent steps. The crystallized product may then be transported to a separate facility, or even possibly to a different country for use in the next reaction, which have associated transportation costs and solvent waste that are not considered in these PMI calculations. However, in telescoped processes, the product can be isolated at the end of the entire process, limiting the number of work up steps required. It can be seen from all reactions that the reaction solvent accounts for over 94% of the total mass used in each process, and any reduction in this will decrease its environmental impact significantly. The PMI calculated for the telescoped optimization is lower than the combined OSAT optimizations due to the concentration of **1** in the reactor being higher, which increased the mass of paracetamol produced in the overall telescoped reaction. This demonstrates the effect that concentration and solvent use have on a process, which is particularly important when scaled up to a kilogram day^–1^ production. Other groups have reported a range of PMIs in continuous processes for API synthesis of the same order of magnitude as the 20% mass of solvent PMI of the telescoped process, showing the benefits of a reduction in solvent use.^49−53^ As important metrics in green chemistry, in the future, PMI and STY would be useful objectives to investigate for these continuous systems.

## Conclusion

A comparison for a single step versus multistep optimization has been described in this report. The BOAEI algorithm was able to efficiently find the optimum conditions, yielding 85% for a two-stage, multiphase reaction. A tube-in-tube separator was employed to ensure complete gas/liquid separation, and multipoint sampling allowed both reaction steps to be sampled so that the effect of all variables could be interpreted from each reaction step. It was found that multistep telescoped reactions, where both steps were optimized simultaneously, lead to a significant reduction in experiments needed to find optimum conditions. This multistep telescoped process benefits from the lack of purification steps needed between reactions, resulting in key improvements of process metrics such as PMI due to a significant decrease in solvent use. It is hoped that this study demonstrates the power of such an approach to reduce waste and encourages widespread implementation during API production moving forward.

To further this work, an investigation into more complicated multiphasic systems would be beneficial as they account for a significant number of reaction steps in the formation of APIs.^54^ Stable heterogeneous catalysts are required for more intense multiobjective optimizations to achieve accurate results at different reaction conditions for a greater number of experiments. A multiobjective optimization would be beneficial to find trade-offs between variables such as yield, or STY, environmental factors such as reaction mass efficiency (RME) or PMI, and process costs. It would be important to account for/adjust optimization protocols for deactivated catalysts during long reactions. One way to address this could be through the implementation of a multibed system, where catalyst beds can be autonomously switched during optimizations to replace spent catalysts. Work on this problem is ongoing within the group. Discrete variable optimizations, optimizing for catalyst amount or type of catalyst, would also provide key process information to minimize precious metal use.

## Supporting Information Available

Materials and methods, photographs, and detailed descriptions of the experimental setups, additional experimental details, all optimization data, example HPLC chromatograms for each optimizations, and a detailed description of the gas/liquid separator (PDF)

## Abbreviations

API: active pharmaceutical ingredient
PAT: process analytical technology
HPLC: high performance liquid chromatography
STY: space-time yield
WHO: World Health Organization
PFA: perfluoroalkoxy alkane
2-MeTHF: 2-methyltetrahydrofuran
OSAT: one-step at a time
BOAEI: Bayesian optimization with adapted expected improvement
LHS: Latin hypercube sampling
IM: intermediate mixture
R1: reactor one
R2: reactor two
PMI: process mass intensity
RME: reaction mass efficiency
