# Supplementary material for: Automated Optimization of a Multistep, Multiphase Continuous Flow Process for Pharmaceutical Synthesis
Source: ACS Sustain Chem Eng. 2024 Oct 3;12(41):15125–33. doi: 10.1021/acssuschemeng.4c05015 (PMC11481092; doi:10.1021/acssuschemeng.4c05015)
Supplement: Supplementary file 1 — sc4c05015_si_001.pdf [file sc4c05015_si_001.pdf]

Supplementary Information

Automated Optimization of a Multi-Stage, Multiphase Continuous Flow Process for Pharmaceutical Synthesis

Sarah L. Boyall †, Holly Clarke†, Thomas Dixon†, Robert W. M. Davidson ≠, Kevin Leslie ‡, Graeme Clemens ‡, Frans L. Muller †, Adam D. Clayton †, Richard A. Bourne†*, and Thomas W. Chamberlain †*

†Institute of Process Research and Development, School of Chemistry, University of Leeds, Leeds, LS2 9JT

‡ Chemical Development, Pharmaceutical Technology & Development, Operations,

AstraZeneca, Macclesfield, SK10 2NA, United Kingdom

≠ Dr. Reddy's Laboratories (EU), 410 Science Park, Milton Road, Cambridge, CB4 0PE United Kingdom

Number of Pages: 21

Number of Figures: 13

Number of Tables: 9

Description: Materials and methods, photographs and detailed descriptions of the experimental setups, additional experimental details, all optimization data, example HPLC chromatograms for each optimization, and a detailed description of the gas/liquid separator.

Table of Contents

Section 1: Reactor Setups and Procedures....S3
Materials and methods S3
Hydrogenation Reactor (R1) setup....S3
Procedure:....S4
Amidation Reactor (R2) setup....S5
Procedure....S5
Combined reactor setup....S8
Procedure....S10
Section 2: Maximum Yield Data S11
Section 3: Optimization Data....S12
Step One S12
Step Two S13
Step Two - IM S14
Telescoped....S15
Section 4: Optimization Plotting....S17
Section 5: Kernel Length Scales....S17
Section 6: Optimum Conditions for each Step S17
Step One Variables and Bounds:....S17
Step Two Variables and Bounds: S17
Telescoped Variables and Bounds: S18
Section 7: Gas-Liquid Separator....S18
Section 8: Online HPLC Analysis S19
Step One S20
Step Two S20
Telescoped....S21
Section 9: Deactivation Data S22
References....S24

Section 1: Reactor Setups and Procedures

Materials and methods

4-Nitrophenol (99%, Fisher), 4-aminophenol (98%, Fluorochem), biphenyl (99%, Merck), acetic anhydride (98%, VWR), acetaminophen (98%, Canyon Chemical), 2-methyl tetrahydrofuran (>=99.5%, Sigma), nitrogen gas (99.998%, BOC) and hydrogen gas (99.99%, BOC) were purchased from suppliers and used without further purification.

Hydrogenation Reactor (R1) setup

Reservoir solutions were prepared and made fully homogeneous by dissolving the desired reagents in solvent under stirring at ambient conditions. Pump 1: 4-Nitrophenol (0.05 M) and biphenyl (0.032 M) in 2-MeTHF (250 mL). Mass Flow Controller: H_2 gas (50 mL min^{-1}). Pump 2: 2-MeTHF (250 mL). Both a Knauer Azura 4.1S dual piston pump and Jasco PU-980 were used to flow in liquid reactants into R1 (1-10 mL min^{-1}). Consistent flow was maintained from both the piston pumps using IDEX P-762 cartridges (75 PSI) on their outlets. A Bronkhorst El-Flow Prestige Mass flow controller (MFC – 5-100 sscm) were used to control gas flow rates into R1. A Jasco BP-2080 plus was used to maintain 40 bar pressure within the system. A Eurotherm temperature controller and aluminum block were used to control the temperature within the system. The catalyst (900 mg 1 wt.% Pd/C Johnson Matthey catalyst pellets product code: 113784) and glass beads (14.95 g) were packed into a stainless-steel column (1/2" OD, 3/8" ID, 0.065" wall thickness), with a 3 mL void volume. Information about the catalyst pellets has been reported by White *et al.* and Boyall *et al.*[1,2] The rest of the setup is made from stainless steel tubing (1/16" OD, 1/32 ID), with a maximum system pressure of 50 bar (limited by the pressure relief valve springs). A Vici EUHA-CI4WE.2 valve was used to sample the reaction

into the online analysis. This analysis was performed by and Agilent 1100 HPLC with an Agilent Poroshell 120 EC-C8 HPLC column with 2.7 μm particle size, L × ID 5 cm × 4.6 mm.

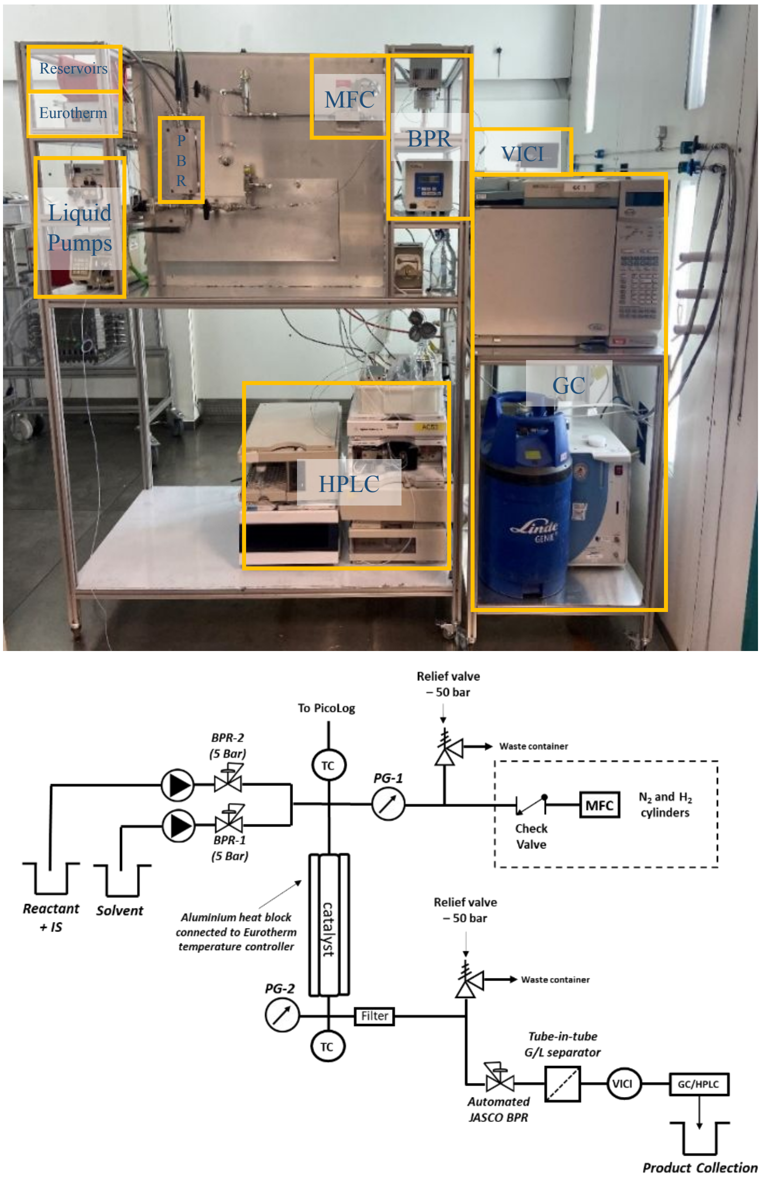

Figure S1. Packed bed reactor used to perform heterogeneous hydrogenation of 4-nitrophenol.

Temperature of column was 35 ˚C, wavelength of VWD was 254 nm, flow rate of 1.5 mL min^{-1}.

Procedure:

1. The system is flushed with nitrogen (50 mL min^{-1}) for 30 minutes.

2. Hydrogen gas (50 mL min^{-1}) is then flowed through the system for 30 minutes.
3. The system is then flushed with 2-MeTHF (0.5 mL min^{-1}) and hydrogen (50 mL min^{-1}) for six hours to activate the catalysts.
4. The 2-MeTHF pump is stopped, and the starting material pump is started (0.5 mL min^{-1}) for two hours to conditions the catalyst bed. The optimization is then started.
5. When the optimization is complete, the entire system is flushed with solvent (1 mL min^{-1}) and nitrogen (50 mL min^{-1}) for one hour.

Amidation Reactor (R2) setup

Reservoir solutions were prepared and made fully homogeneous by stirring under ambient conditions. Pump 1: 4-Aminophenol (0.05 M) and biphenyl (0.032 M) in 2-MeTHF (250 mL). Pump 2: Acetic anhydride (0.4 M) in 2-MeTHF (250 mL). Pump 3: 2-MeTHF (250 mL). Jasco P-980 pumps were used to control liquid flow rates in the system and a 100 PSI IDEX BPR cartridge was used to maintain pressure. The reactor system was made up of 0.5 mL Polyfon PFA tubing of length 1.01 m (1/16” OD, 1/32” ID) and was coiled around an aluminum heating block, connected to a Eurotherm temperature controller. A Vici EUHA-CI4WE.2 sampling valve was used to sample the reaction into the online analysis. This analysis was performed by and Agilent 1100 HPLC fitted with an Agilent Poroshell 120 EC-C8 HPLC column with 2.7 μm particle size, L × ID 5 cm × 4.6 mm. Temperature of column was 35 ˚C, wavelength of DAD was 254 nm, flow rate of 1.5 mL min^{-1}.

Procedure

1. The system is flushed with IPA for 1 minute at 1 mL min^{-1} per pump.

2. The system is then flushed with solvent (2-MeTHF) for 2 minutes at 1 mL min^{-1} per pump.
3. The pumps are connected to their respective reagents and starting material reservoirs and flushed through the system for 2 minutes at 1 mL min^{-1} per pump.
4. The Optimization is started.
5. When optimization is complete, the entire system is flushed with solvent again for 2 minutes at 1 mL min^{-1} per pump.

6. The system is finally flushed with IPA for 2 minutes at 1 mL min^{-1} per pump and left under

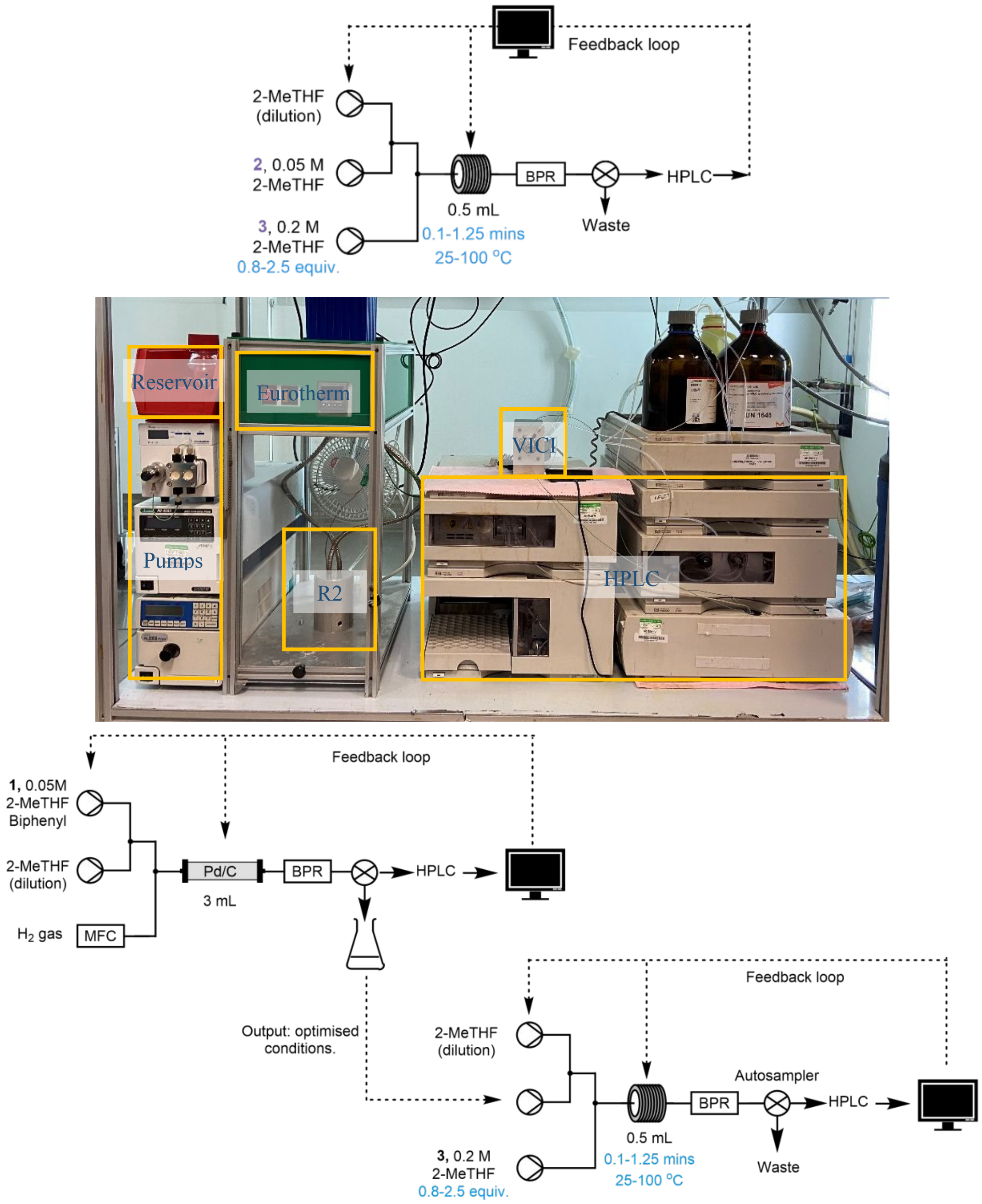

IPA to maintain the pump health.

Figure S2. Top: Flow reactor used to perform single-step amidation reaction of 4-aminophenol for the OSAT amidation reaction of 4-aminophenol to paracetamol, Middle: Image of R2. Bottom: Flow reactor used to perform single-step amidation reaction of 4-aminophenol for the step two-IM amidation reaction of 4-aminophenol to paracetamol

Combined reactor setup

Reservoir solutions were prepared by dissolving the desired reagents in solvent under stirring at ambient conditions. Pump 1: 4-Nitrophenol (0.05 M) and biphenyl (0.032 M) in 2-MeTHF (250 mL). Mass Flow Controller: H_2 gas (50 mL min^{-1}). Pump 2: Acetic anhydride (0.4 M) in 2-MeTHF (250 mL). Pump 3: 2-MeTHF (250 mL). Reservoir solutions were replenished when required.

Reactor 1 was an aluminium block fitted with stainless steel tubing (1/2" OD, 3/8" ID, 0.065" wall thickness) with 10.8 mL volume when empty, and 4 mL void volume when packed with 12.52 g glass beads (2 mm diameter) and 1.501 g 1% Pd/C JM catalyst pellets and maintained under 40 bar (580 psi) of pressure using a Jasco BP-2080 plus back pressure regulator. The rest of the tubing was stainless steel (1/16" OD, 1/32 ID). A Knauer 4.1S dual piston pump and a Bronkhorst El-Flow Prestige Mass Flow Controller were used to pump liquids and gases.

Reactor 2 was a cylindrical aluminium block with 1.5 mL Polyfon PFA tubing coiled (1/16" OD, 1/32" ID) and maintained under 100 psi of pressure with a IDEX BPR cartridge, controlled by a Eurotherm temperature controller. The pumps used were a Jasco P-980 dual piston pump and a Knauer 4.1S dual piston pump.

Two VICI Valco EUDA-CI4W sample loops (4-port) with 0.2 µL rotors were used for online sampling and monitoring of each step.[3] The 100 psi BPR on the outlet of the second reactor was used in tandem with a second 100 psi BPR and a needle valve on the tube-in-tube separator gas outlet. The needle valve was tightened and loosened as needed to ensure optimal separation of the gas from the flow stream before passing into reactor 2. See section 3 for more details.

Online analysis was performed with a HP Agilent 1100 HPLC System fitted with an Agilent Poroshell 120 EC-C8 HPLC column with 2.7 µm particle size, L × ID 5 cm × 4.6 mm. The method gradient is shown in

Table **S1** and Figure S4. It is made up of a 6-minute method which is doubled, where the autosampler after reactor 1 is fired at 0 minutes, and the autosampler after reactor two is fired at 6 minutes. Temperature of column was 35 °C, wavelength of VWD was 254 nm, flow rate of 1.5 mL min^{-1}.

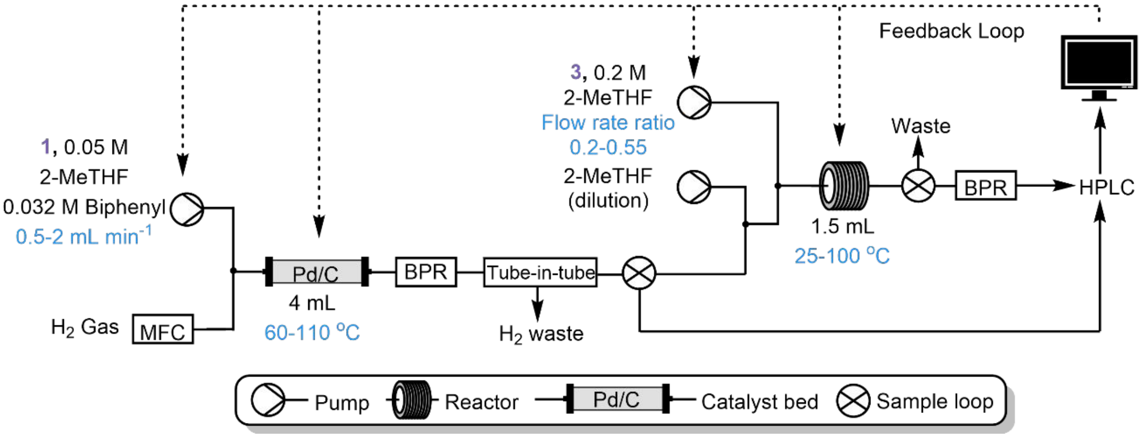

Figure S3. Continuous flow reactor set-up for the telescoped two-step synthesis of paracetamol from 4-nitrophenol.

Table S1. HPLC method used in the 4-aminophenol amidation reaction, where % B is the percentage of MeCN.

Time / min	% B
**0**	5
**1**	5
**2.5**	95
**4.5**	95
**5**	5
**6**	5
**7**	5
**8.5**	95
**10.5**	95
**11**	5
**12**	5

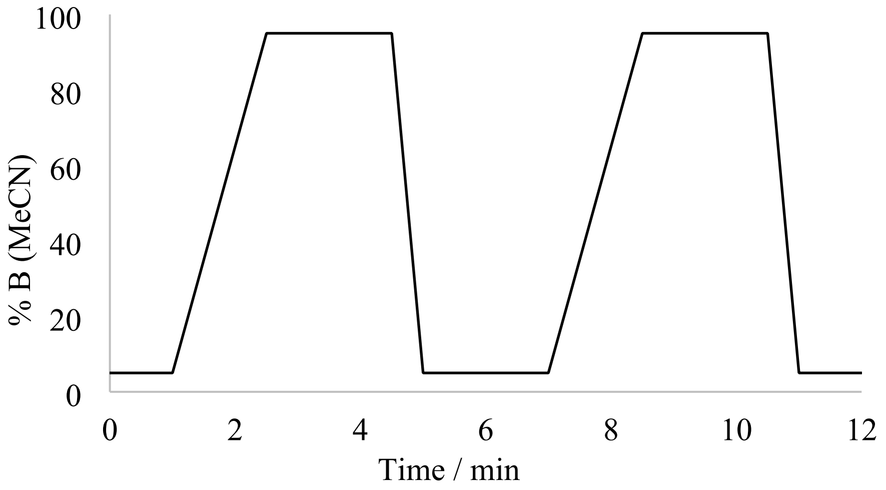

Figure S4. HPLC method gradient for the two-step telescoped hydrogenation and amidation reaction.

Procedure

1. R1 system is flushed with nitrogen gas (50 mL min^{-1}) for 30 minutes.
2. R1 system is flushed with hydrogen gas (50 mL min^{-1}) for 30 minutes.
3. R1 system is flushed with 2-MeTHF (0.5 mL min^{-1}) and hydrogen (50 mL min^{-1}) for six hours.
4. The 2-MeTHF pump is stopped, and the starting material pump is started (0.5 mL min^{-1}) for two hours.
5. R1 and R2 are connected via the tube-in-tube gas/liquid separator, all pumps are set to minimum flow rates and temperatures, and the optimization is then started.
6. When the optimization is complete, the entire system is flushed with solvent (1 mL min^{-1}) and nitrogen (50 mL min^{-1}) for one hour.

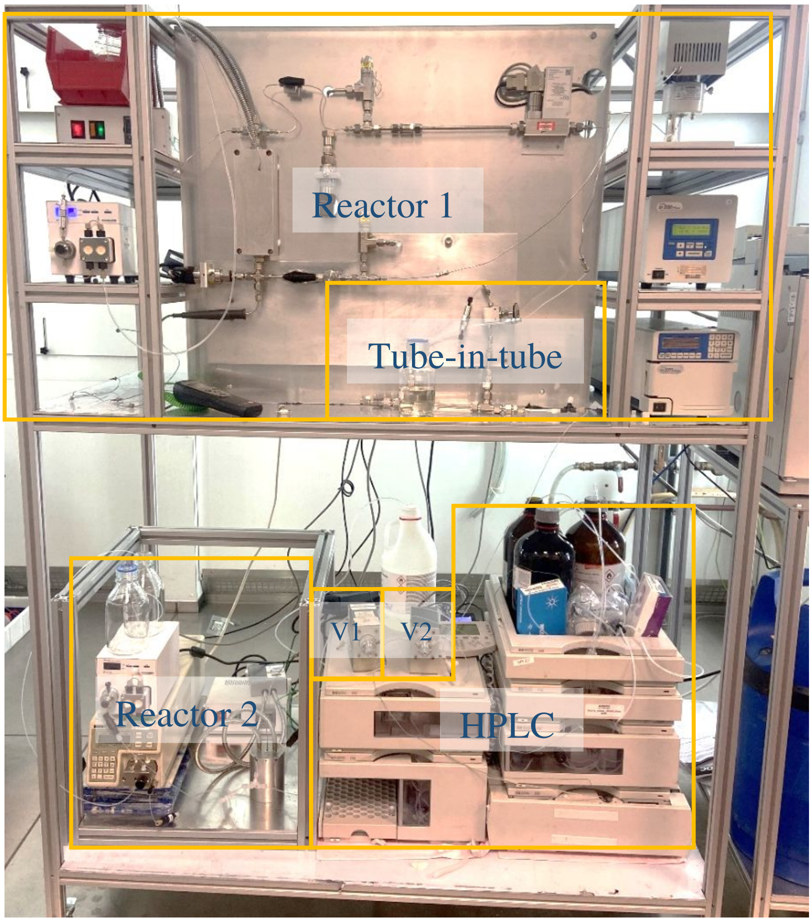

Figure S5. – Reactor setup for combined telescoped two-step synthesis of paracetamol. R1 – reactor 1 heterogeneous packed bed reactor, R2 – PFA plug flow coiled reactor, V1 – vici sampling valve 1 for R1, and V2 – vici sampling valve for R2.

Section 2: Maximum Yield Data

All data was plotted on a single graph showing the number of the experiment and maximum yield achieved chronologically in each optimization.

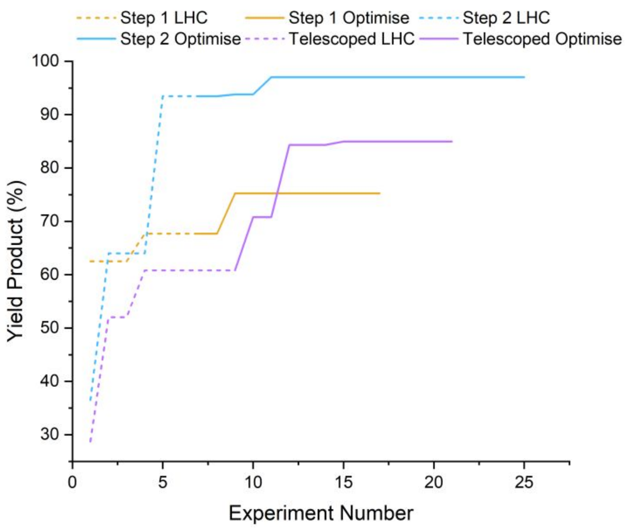

Figure S6. Comparison of experiment number and maximum yield achieved chronologically in each optimization.

Section 3: Optimization Data

Optimization data for the four single objective optimizations are outlined in Table S2, Table S3, Table S4, and Table S5.

Step One

Table S2. Optimization data for OSAT step one single objective optimization.

Exp. No.	Exp. Type	Variable			Flow Rate / mL min^{-1}		Objective
		Liq. Flow Rate / mL min^{-1}	Temp. / °C	Conc. / M	4-Nitrophenol	Solvent	Yield / %
**1**	Initial	0.685	68.1	0.030	0.414	0.270	43.25
**2**	Initial	1.422	44.6	0.019	0.538	0.881	20.52
**3**	Initial	0.965	62.9	0.045	0.874	0.091	30.42
**4**	Initial	0.739	52. 8	0.044	0.648	0.092	31.82
**5**	Initial	0.880	78.7	0.038	0.664	0.214	41.17
**6**	Initial	0.521	58.2	0.026	0.272	0.248	44.68
**7**	Initial	0.594	77.9	0.013	0.160	0.434	56.63
**8**	Refinement	0.508	77.1	0.029	0.290	0.218	59.22
**9**	Refinement	0.502	85.0	0.010	0.100	0.400	64.58
**10**	Refinement	0.503	85.0	0.011	0.101	0.399	62.33
**11**	Refinement	0.502	84.9	0.010	0.100	0.400	63.30
**12**	Refinement	0.500	85.0	0.049	0.488	0.012	54.40
**13**	Refinement	0.508	85.0	0.010	0.102	0.407	63.20
**14**	Refinement	0.500	84.0	0.010	0.100	0.400	59.00

Step Two

Table S3. Optimization data for OSAT step two single objective optimization.

Exp No.	Exp. Type	Variables			Flow Rate / mL min^{-1}			Objective
		Res. Time / min	Equiv.	Temp. / °C	4-Aminophenol	Acetic Anhydride	Solvent	Yield / %
1	Initial	0.65	1.33	27.5	0.308	0.102	0.360	36.48
2	Initial	0.95	1.97	39.0	0.211	0.104	0.213	64.00
3	Initial	0.28	2.09	53.7	0.727	0.379	0.711	47.76
4	Initial	0.53	1.26	63.8	0.378	0.119	0.448	48.56
5	Initial	0.80	2.43	78.2	0.251	0.153	0.224	93.44
6	Initial	1.14	1.04	79.1	0.176	0.045	0.218	66.94
7	Initial	0.14	1.76	95.3	1.406	0.619	1.491	39.16
8	Refinement	0.91	2.47	50.2	0.220	0.135	0.194	91.02
9	Refinement	0.83	2.22	99.5	0.241	0.134	0.228	93.81
10	Refinement	0.68	2.50	68.3	0.296	0.185	0.259	82.02
11	Refinement	1.19	2.20	100.0	0.167	0.092	0.159	97.03
12	Refinement	1.00	2.50	95.9	0.200	0.125	0.175	96.17
13	Refinement	1.12	2.41	73.5	0.179	0.108	0.161	94.88
14	Refinement	0.95	2.50	79.8	0.211	0.132	0.185	96.28
15	Refinement	1.25	2.16	40.9	0.160	0.086	0.154	84.71
16	Refinement	0.99	2.29	73.8	0.202	0.116	0.187	94.36
17	Refinement	1.25	1.70	100.0	0.160	0.068	0.172	96.94
18	Refinement	1.25	2.50	87.2	0.160	0.100	0.140	95.90
19	Refinement	0.10	0.80	100.0	1.990	0.398	2.587	13.66
20	Refinement	0.95	2.50	80.8	0.211	0.132	0.185	89.90
21	Refinement	1.01	2.50	81.6	0.197	0.123	0.173	91.32
22	Refinement	1.01	2.50	80.3	0.198	0.124	0.173	88.63
23	Refinement	1.00	2.50	89.6	0.200	0.125	0.175	88.90
24	Refinement	1.02	2.50	77.6	0.196	0.122	0.171	91.88
25	Refinement	1.01	2.50	80.3	0.199	0.124	0.174	91.71

Step Two - IM

Table S4. Optimization data for OSAT step two-IM single objective optimization.

Exp No.	Exp. Type	Variables			Flow Rate / mL min^{-1}			Objective
		**Res. Time / min**	**Equiv.**	**Temp. / °C**	**4-Aminophenol**	**Acetic Anhydride**	**Solvent**	**Yield / %**
1	Initial	0.65	1.33	27.5	0.308	0.102	0.360	38.74
2	Initial	0.95	1.97	39.0	0.211	0.104	0.213	79.98
3	Initial	0.28	2.09	53.7	0.727	0.379	0.711	49.63
4	Initial	0.53	1.26	63.8	0.378	0.119	0.448	52.03
5	Initial	0.80	2.43	78.2	0.251	0.153	0.224	95.17
6	Initial	1.14	1.04	79.1	0.176	0.045	0.218	74.81
7	Initial	0.14	1.76	95.3	1.406	0.619	1.491	41.88
8	Refinement	0.88	2.50	80.3	0.226	0.141	0.198	95.36
9	Refinement	0.82	2.04	98.7	0.245	0.125	0.243	94.42
10	Refinement	0.87	2.50	62.1	0.229	0.143	0.200	93.23
11	Refinement	1.21	2.50	28.6	0.165	0.103	0.144	83.17
12	Refinement	1.14	0.80	48.1	0.176	0.035	0.228	37.29
13	Refinement	0.97	2.05	76.5	0.205	0.105	0.203	91.22
14	Refinement	0.73	2.50	91.5	0.274	0.171	0.239	96.43
15	Refinement	1.25	2.50	91.0	0.160	0.100	0.140	97.18
16	Refinement	1.20	2.50	80.7	0.167	0.105	0.146	97.77
17	Refinement	1.16	2.50	80.8	0.173	0.108	0.151	97.73
18	Refinement	1.12	2.50	82.1	0.179	0.112	0.156	98.52
19	Refinement	1.25	2.01	100.0	0.160	0.080	0.160	98.83
20	Refinement	1.13	2.50	83.7	0.177	0.110	0.155	97.62
21	Refinement	1.14	2.50	100.0	0.175	0.109	0.153	98.71
22	Refinement	1.12	2.20	91.1	0.179	0.098	0.170	98.42
23	Refinement	1.25	2.37	51.4	0.160	0.095	0.146	88.93
24	Refinement	1.25	2.50	58.5	0.160	0.100	0.140	94.84
25	Refinement	1.25	2.50	46.8	0.160	0.100	0.140	90.88

Telescoped

Table S5. Optimization data for telescoped single objective optimization.

Exp No.	Exp. Type	Variables				Equivalents Acetic Anhydride	Flow Rate / mL min^{-1}		Yield (step 1) / %	Objective
		Nitrophenol / mL min^{-1}	Temp. R1 / °C	Temp. R2 / °C	Flow Rate Ratio		Acetic Anhydride	Solvent		Yield (Step 2) / %
**1**	Initial	1.44	60.6	44.9	0.29	1.16	0.418	0.542	54.3	28.3
**2**	Initial	0.63	67.5	91.4	0.36	1.44	0.225	0.193	68.3	52.0
**3**	Initial	0.91	76.1	31.7	0.52	2.06	0.471	0.138	62.6	40.8
**4**	Initial	0.67	80.4	94.9	0.47	1.87	0.312	0.134	68.8	60.8
**5**	Initial	1.09	85.0	77.1	0.21	0.83	0.226	0.498	60.7	37.7
**6**	Initial	1.56	89.0	54.2	0.27	1.10	0.428	0.614	52.1	32.0
**7**	Initial	1.22	94.9	71.0	0.35	1.38	0.421	0.392	62.2	51.5
**8**	Initial	1.93	99.4	34.1	0.42	1.68	0.813	0.477	56.1	28.8
**9**	Initial	1.83	106.1	59.5	0.48	1.93	0.883	0.335	64.7	39.9
**10**	Refinement	1.01	109.9	83.2	0.46	1.85	0.468	0.206	78.6	70.8
**11**	Refinement	1.12	109.0	91.7	0.52	2.09	0.586	0.160	71.5	65.1
**12**	Refinement	0.50	110.0	67.0	0.55	2.20	0.275	0.058	86.8	84.6
**13**	Refinement	0.71	110.0	77.1	0.55	2.20	0.389	0.082	83.9	82.1
**14**	Refinement	0.50	110.0	90.4	0.20	0.80	0.100	0.233	82.0	59.0
**15**	Refinement	0.50	110.0	32.2	0.48	1.92	0.240	0.093	83.3	85.0
**16**	Refinement	0.50	110.0	64.0	0.43	1.72	0.214	0.119	83.6	79.6
**17**	Refinement	0.56	110.0	80.0	0.55	2.20	0.310	0.066	80.0	76.8
**18**	Refinement	0.67	110.0	25.0	0.55	2.20	0.368	0.078	76.8	71.5

Section 4: Optimization Plotting

All 4/5D optimization graphs were plotted using plotly, see link to view plotting code: https://github.com/Bourne-Group/TelescopedOptimisationPlotlyGraphs.

Section 5: Kernel Length Scales

Kernal length scales were calculated for each optimization variable during the OSAT, IM and telescoped optimizations and are shown in Figure S7.

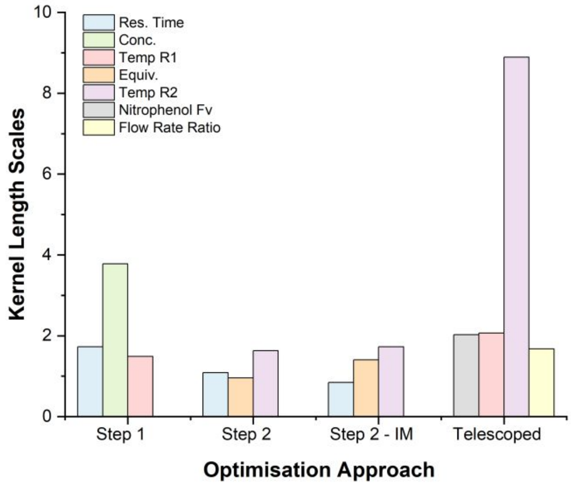

Figure S7. Comparison of optimization variables kernel length scales, showing their importance to the overall reaction output.

Section 6: Optimum Conditions for each Step

Step One Variables and Bounds:

Temperature – 40 – 85 ˚C

Concentration – 0.01 – 0.05 M

Residence Time – 0.5 – 1.5 mL min^{-1}

Step Two Variables and Bounds:

Temperature – 25 – 100 °C

Equivalents – 0.8 – 2.5

Residence Time – 0.1 – 1.25

Telescoped Variables and Bounds:

Temperature R1 – 60 – 110 ˚C

4-Nitrophenol Flow Rate – 0.5 – 2 mL min^{-1}

Flow Rate Ratio – 0.2 – 0.55 (0.8 – 2.4 equivalents acetic anhydride)

Temperature R2 – 30 – 100 ˚C

Step Number	Temp. R1 / ˚C	4-Nitrophenol flow rate / mL min^{-1}	Conc. / M	Equiv.	Temp. R2 / ˚C	Res. Time / min
OSAT - 1	85	0.5	0.01	-	-	-
OSAT - 2	-	-	-	2.2	100	1.19
2 – SM Res from step 1	-	-	-	2.5	100	1.14
Telescoped	110	0.5	-	2.4	66.96	-

Table S6. Summary of Optimum conditions for all single objective optimizations.

Section 7: Gas-Liquid Separator

A tube-in-tube separator was used to perform gas liquid separation. The ePTFE tubing (OD 0.25", ID 0.118", density of 1.15 g cm^{-3}) was encased in a stainless steel tube as is described in more detail by Harding *et al.*[4] The separator was adapted to add an IDEX 100 PSI cartridge BPR was added after the needle valve to equal the pressure of the downstream reactor (R2). A needle valve on the gas outlet was used to adjust the back pressure applied to reaction stream outside of the porous ePTFE tubing. This was adjusted at the start of the optimization to allow complete phase separation of the gas and liquid phases.

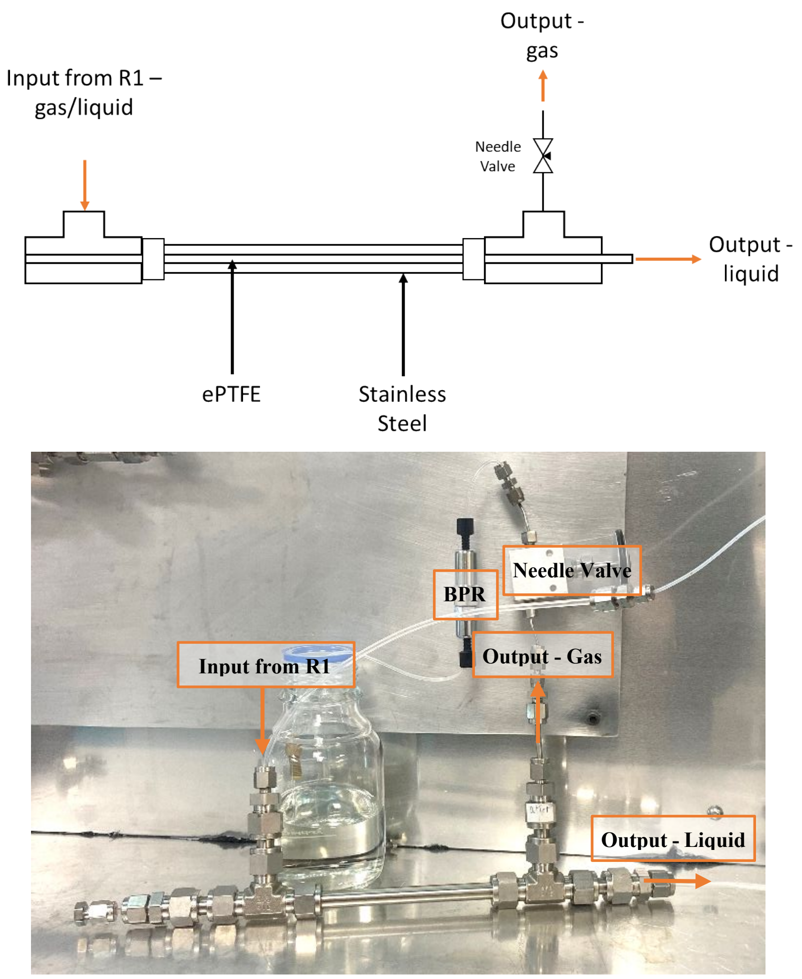

Figure S8. Diagrams showing the tube-in-tube separator.

Section 8: Online HPLC Analysis

Example chromatograms for step one, step two and the telescoped multistep optimizations are shown below.

Step One

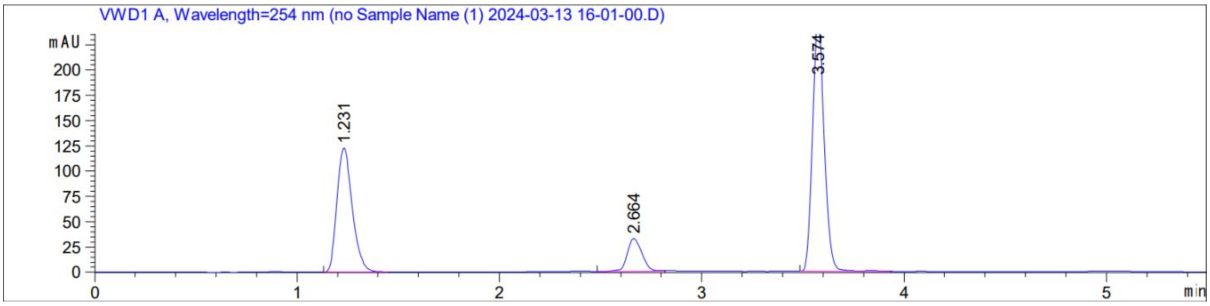

Figure S9. Example HPLC chromatogram for the hydrogenation optimization of 4-aminophenol.

Table S7 - Peaks picking for Figure S9 in hydrogenation optimization identifying the product (P), starting material (SM) and internal standard (IS).

Peak position / min	Peak identification
**1.231**	4-Aminophenol (P)
**2.664**	4-Nitrophenol (SM)
**3.574**	Biphenyl (IS)

Step Two

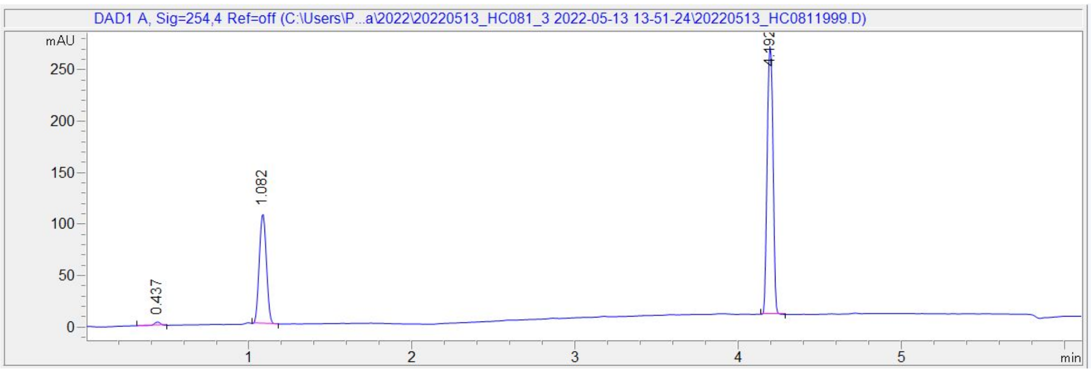

Figure S10. Example HPLC chromatogram for the amidation optimization of paracetamol.

Table S8. Peaks picking for Figure S10 in amidation optimization identifying the product (P), starting material (SM) and internal standard (IS).

Wavelength / nm	Peak position / min	Peak identification
**254**	0.4	4-Aminophenol (SM)
**220**	1.1	Paracetamol (P)
**220**	4.2	Biphenyl (IS)

Telescoped

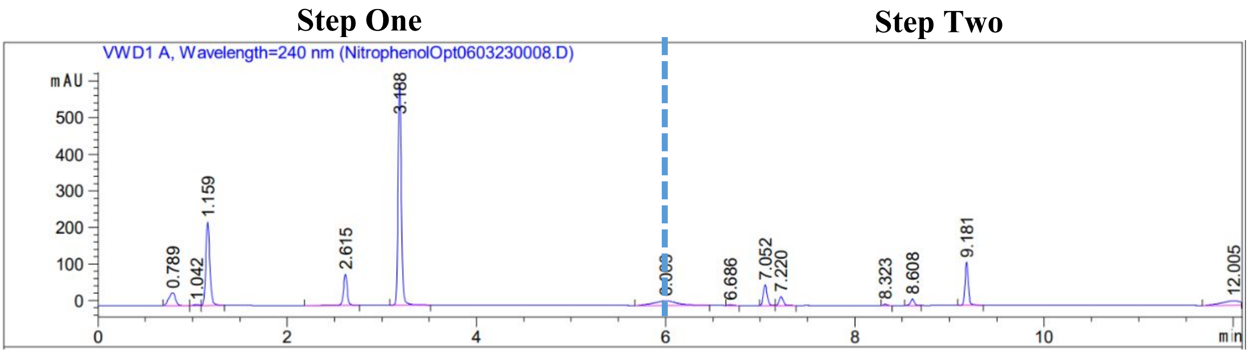

Figure S11. Example HPLC chromatogram for the telescoped hydrogenation-amidation optimization of paracetamol where 0-6 minutes is the first sample from step one and 6-12 minutes is the second sample from step two.

Table S9. Peaks picking for Figure S11 in the telescoped hydrogenation-amidation optimization of paracetamol optimization identifying the product from step one (P1), starting material from step one (SM1), internal standard (IS), product from step two (P2), and by-product from step two (BP2).

Step/ Sample	Peak position / min	Peak identification
**1**	0.79	4-Aminophenol (P1)
**1**	1.16	Impurity
**1**	2.62	4-Nitrophenol (SM1)
**1**	3.19	Biphenyl (IS)
**1**	6.00	Solvent
**2**	6.69	4-Aminophenol (P1)
**2**	7.05	Impurity
**2**	7.22	Paracetamol (P2)
**2**	8.32	Diacetamate (BP2)
**2**	8.61	4-Nitrophenol (SM1)
**2**	9.18	Biphenyl (IS)
**2**	12.00	Solvent

Section 9: Deactivation Data

Single step deactivation profile for two-day optimization:

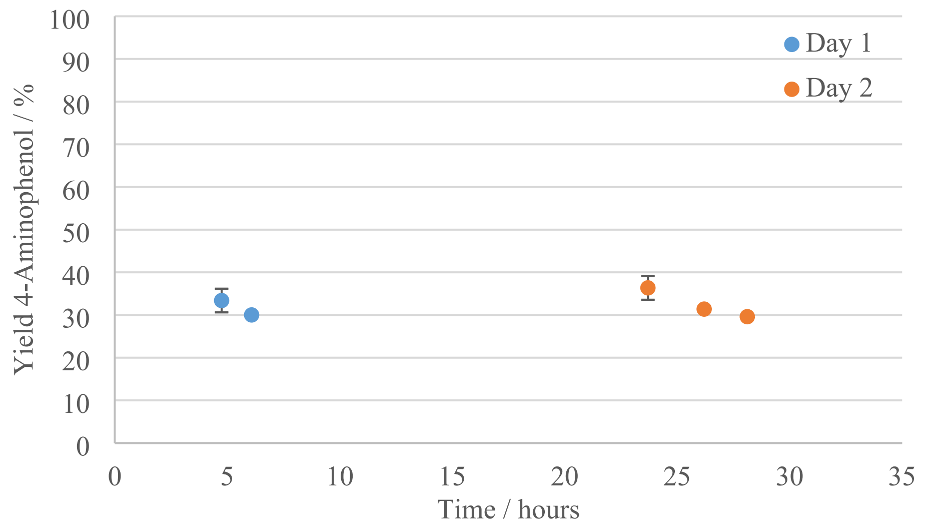

Figure S12. Deactivation data for step one, a 2-day optimization.

Telescoped step one deactivation profile for three-day optimization – experiments after 55 hours discounted, and optimization stopped.

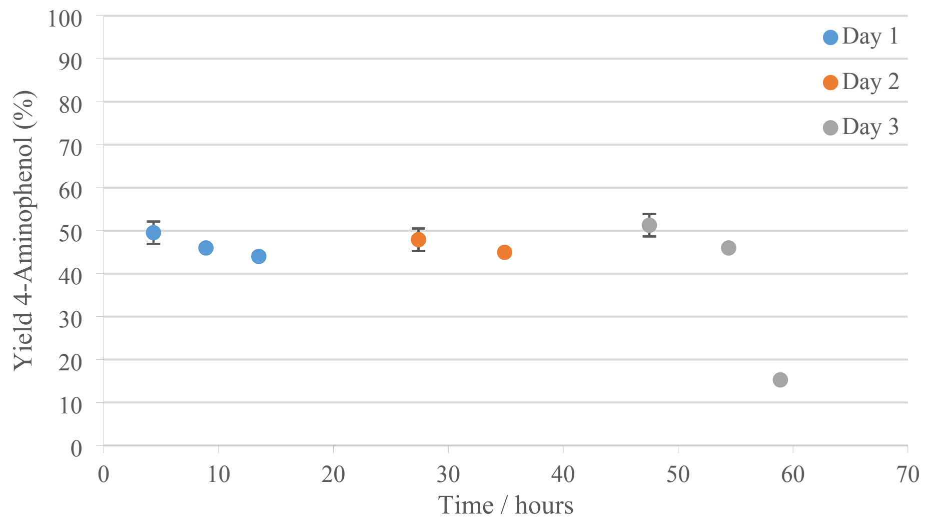

Figure S13. Deactivation data for telescoped optimization over 3 days of experiments.

References

(1) White, J. P., Chamberlain, T. W., Bourne, R. A., Taylor, D., Brennan, C., Muller, F. L. Decoupling the Relative Rate of Hydrogen Uptake via Convection and Mass Transfer by a Single Catalytic Pellet in a Scaled down Trickle Bed Reactor. *Chem. Eng. J.* **2020**, *394*, 124290. DOI: 10.1016/j.cej.2020.124290

(2) Boyall, S. L., Berman, P., Griffiths, A., Massey, A., Dixon, T., Shaw, T., Miller, J., White, J. P., Menzel, R., Leslie, K., Clemens, G., Muller, F. L., Bourne, R. A., Chamberlain, T. W. Palladium Nanoparticle Deposition on Spherical Carbon Supports for Heterogeneous Catalysis in Continuous Flow. *Catal. Sci. Technol.* **2024**, *14*, 2563-2573. DOI: 10.1039/D3CY01718D

(3) Clayton, A. D. Pyzer-Knapp, E. O. Purdie, M. Jones, M. F. Barthelme, A. Pavey, J. Kapur, N. Chamberlain, T. W. Blacker, A. J. Bourne, R. A. Bayesian Self-Optimization for Telescoped Continuous Flow Synthesis. *Angew. Chem. Int. Ed.* **2023**, *62* (3). DOI: 10.1002/ANIE.202214511.

(4) Harding, M. J.; Feng, B.; Lopez-Rodriguez, R.; O'Connor, H.; Dowling, D.; Gibson, G.; Girard, K. P.; Ferguson, S. Concentric Annular Liquid–Liquid Phase Separation for Flow Chemistry and Continuous Processing. *React. Chem. Eng.* **2021**, *6* (9), 1635–1643. DOI: 10.1039/D1RE00119A.
